# Morphologic and Molecular Characterization of a *Demodex* (Acari: Demodicidae) Species from White-Tailed Deer (*Odocoileus virginianus*)

**DOI:** 10.5402/2013/342918

**Published:** 2013-01-15

**Authors:** Michael J. Yabsley, Sarah E. Clay, Samantha E. J. Gibbs, Mark W. Cunningham, Michaela G. Austel

**Affiliations:** ^1^Southeastern Cooperative Wildlife Disease Study, Department of Population Health, The University of Georgia College of Veterinary Medicine, Wildlife Health Building, Athens, GA 30602, USA; ^2^Warnell School of Forestry and Natural Resources, The University of Georgia, Athens, GA 30602, USA; ^3^Division of Migratory Bird Management, U.S Fish & Wildlife Service, Laurel, MD 20708, USA; ^4^Florida Fish and Wildlife Conservation Commission, Gainesville, FL 32653, USA; ^5^Department of Small Animal Medicine and Surgery, The University of Georgia College of Veterinary Medicine, University of Georgia, Athens, GA 30602, USA; ^6^Massachusetts Veterinary Referral Hospital, Woburn, MA 01801, USA

## Abstract

*Demodex* mites, although usually nonpathogenic, can cause a wide range of dermatological lesions ranging from mild skin irritation and alopecia to severe furunculosis. Recently, a case of demodicosis from a white-tailed deer (*Odocoileus virginianus*) revealed a *Demodex* species morphologically distinct from *Demodex odocoilei*. All life cycle stages were considerably larger than *D. odocoilei* and although similar in size to *D. kutzeri* and *D. acutipes* from European cervids, numerous morphometrics distinguished the four species. Adult males and females were 209.1 ± 13.1 and 225.5 ± 13.4 *μ*m in length, respectively. Ova, larva, and nymphs measured 65.1 ± 4.1, 124.9 ± 11.6, and 205.1 ± 19.4 *μ*m in length, respectively. For phylogenetic analyses, a portion of the 18S rRNA gene was amplified and sequenced from samples of the WTD *Demodex* sp., two *Demodex* samples from domestic dogs, and *Demodex ursi* from a black bear. Phylogenetic analyses indicated that the WTD *Demodex* was most similar to *D. musculi* from laboratory mice. A partial sequence from *D. ursi* was identical to the WTD *Demodex* sequence; however, these two species can be differentiated morphologically. This paper describes a second *Demodex* species from white-tailed deer and indicates that 18S rRNA is useful for phylogenetic analysis of most *Demodex* species, but two morphologically distinct species had identical partial sequences. Additional gene targets should be investigated for phylogenetic and parasite-host association studies.

## 1. Introduction

Mites of the genus *Demodex* are commonly found in the hair follicles and sebaceous glands of most mammals. In general, *Demodex* are considered to be host-species specific and some hosts can be infested with two or more distinct species (e.g., *D. canis*, *D. injai*, an undescribed short form *Demodex* sp. in dogs, *D. brevis* and *D. folliculorum *in humans, *D. odocoilei *in white-tailed deer (*Odocoileus virginianus*), and *D. bovis* in cattle) [1–5]. In general, *Demodex *infestations can vary widely in clinical presentation (from asymptomatic animals to cases with variable extends of alopecia, varying degrees of thickening of the skin, to cutaneous nodular lesions and severe dermatitis/furunculosis). In small animals, some animals have concurrent immunosuppression; however, it is unclear what role immunosuppression plays in generalized clinical demodicosis. In addition, some individuals or species, especially cervids in Europe, as well as cattle, develop a nodular demodicosis [3, 6]. In a longitudinal study of cattle, these nodules waxed and waned both in number and size over time [6]. 

A *Demodex *sp. was first detected in a white-tailed deer from Georgia in the 1960s [7]. This infested deer exhibited hair loss and thickening of the skin on the head, neck, and shoulders. A second incidence of demodicosis was reported in Oklahoma in 1971 from a white-tailed deer with alopecia [8]. *Demodex odocoilei*, currently the only known *Demodex* sp. from white-tailed deer in the United States, was described using material from skin scrapings of deer in Georgia, Virginia, and Oklahoma [4]. These mites were obtained via deep skin scrapings of formalin-fixed tissue. Histology of the skin samples did indicate hair loss and distention of the hair follicle and sebaceous glands, but the presence of mites was not associated with inflammation [4]. Mites morphologically consistent with *D. odocoilei *were reported from Columbian black-tailed deer (*Odocoileus hemionus columbianus*) and mule deer (*Odocoileus hemionus* hemionus) from the western United States and Canada [9, 10]. Recently, *Demodex kutzeri, *a species of European cervids, was reported from three species of cervids (one mule deer and one Rocky Mountain elk (*Cervus elaphus nelsoni*) from Colorado and a white-tailed deer from South Dakota) as well as a white-tailed deer from South Carolina (since 1971) [5]. Throughout the southeastern United States, numerous other cases of demodicosis have been diagnosed in white-tailed deers (SCWDS, unpublished data); however, the causative species of *Demodex* was not determined.

*Demodex* is currently classified in the subclass Acari, superorder Acariformes, order Prostigmata, superfamily Chelyetoidea, and family Demodicidae. Although PCR and sequence analysis have served as vital techniques to investigate relationships of numerous organisms, including mites, there is currently only genetic information available for four *Demodex *species, *D. folliculorum* and *D. brevis* from humans, *D. canis *from a dog [11], and *D. musculi* from mice in Genbank (accession number JF834894). The increased availability of sequence data would allow researchers to investigate the diversity of *Demodex* species infecting different or the same host species, host specificity, and investigate any geographic variability among *Demodex* (e.g., *D. canis* from various continents).

 A sample of *Demodex* from a white-tailed deer clinical case submitted to the Southeastern Cooperative Wildlife Disease Study (SCWDS) was found to differ morphologically from *D. odocoilei* and other *Demodex species* reported from cervids. In this study, our goal was to characterize this *Demodex* sp. using a combination of morphological and molecular techniques. The morphologic characters of this *Demodex* sp. were compared to other cervid *Demodex* including *D. odocoilei*, *D. acutipes*, and *D. kutzeri* [3, 4, 12]. For our molecular analysis, we compared the *Demodex* sp. from the white-tailed deer with two *Demodex *samples from dogs, *Demodex ursi* from a black bear, and related sequences available in GenBank from *Demodex* and related mites.

## 2. Materials and Methods

In October 2002, a 2.5-year-old female hunter-killed deer from Lee County, South Carolina (USA) was submitted to SCWDS for diagnostic evaluation because of grossly visible skin lesions. A complete necropsy was performed and samples of skin were fixed in 10% buffered formalin, embedded in paraffin, sectioned, and stained with hematoxylin and eosin. Other portions of skin were preserved in 100% ethanol for PCR analysis.

*Demodex* mites removed from nodules were placed in immersion oil and covered with a cover slip. Mites were examined on 400X power and measurements of all life stages (adult male and female, nymph, larva, and ovum) were taken using a calibrated micrometer. Measurement of the adults included total body length, gnathosomal length, podosomal length, opisthosomal length, and presence of and length of aedeagus or vulva as appropriate. Measurements of ova, larvae, and nymphs included total length and width for ova. A total of 38 ova, 38 larvae, 40 nymphs, 42 female, and 44 male mites were measured. The Average and standard deviation for each measurement category were calculated.

 Samples for molecular characterization were collected from various sources. Skin nodules from the white-tailed deer clinical case had been preserved in 100% ethanol, skin samples of *D. ursi* from a black bear from Florida (USA) with generalized demodicosis were stored at −20°C until analysis, and samples of *D. canis* and a short form *Demodex* sp. from dogs from Clarke County, Georgia (USA) were obtained as fresh skin scrapings. Each individual sample was placed into a Sarstedt O-ring tube with two copper BBs. The sample was macerated for 2 one-minute cycles at 1/2-speed setting with a Mini Beadbeater 8 (Biospec Products, Bartlesville, OK). Phosphate-buffered saline (200 *μ*L) was added to each tube and the samples were vortexed thoroughly. DNA was extracted using the GFX Genomic Blood DNA Purification Kit (Amersham Biosciences, Piscataway, NJ, USA) according to the manufacturer's directions for direct blood. Because the primers were designed for ticks, we used three tick species as positive controls. DNA was extracted from three individual ticks (*Dermacentor variabilis*, *Amblyomma americanum*, and *Haemaphysalis leporispalustris*) as described for the mites except that they were individually frozen in liquid nitrogen before being macerated. Water controls were included in each step of the PCR analyses (extraction, primary reaction, and secondary reaction) to serve as negative controls.

 Overlapping primer pairs that amplify the 18S rRNA gene of the ticks and other mites were selected to provide near full-length sequence data for *Demodex* [13]. Primary reactions were conducted with primers NS1/NS8 and NS1/NS4. Overlapping nested and heminested secondary PCR reactions with primer pairs NS1/NS2, NS12+/NS2, NS1/NS4, and NS58.1/NS8 were conducted as described [13]. Briefly, each 25 *μ*L reaction contained 11 *μ*L molecular biology grade water, 2.5 *μ*L 25 mM MgCl_2_, 5 *μ*L 5X colorless buffer (Promega, Madison, WI), 0.25 *μ*L of 20 mM dNTPs (Promega), 0.5 *μ*L each primer (50 *μ*M), and 0.25 *μ*L GoTaq Flexi (Promega). Reaction conditions consisted of 1 min at 92°C followed by 10 cycles of 1 min at 92°C, 1 minute at 48°C, and 90 sec at 72°C, then 32 cycles of 1 min at 92°C, 35 sec at 54°C, and 90 sec at 72°C, and a final extension time of 7 min at 72°C. Amplicons were visualized in a 2% agarose gel stained with ethidium bromide. Amplicons were purified using the QIAquick Gel Purification Kit (Qiagen, Valencia, CA, USA) and bidirectionally sequenced at the Integrated Biotechnology Laboratories (University of Georgia, Athens, GA, USA).

Sequences obtained from this study and from related organisms stored in GenBank were aligned using the multisequence alignment Clustal algorithm in MEGA version 3.1 [14]. GenBank accession numbers for the *Demodex* sp. from white-tailed deer, *D. canis*, the short form *Demodex* sp. from a dog, and *D. ursi *are KC010483, KC010485, KC010484, and KC010482, respectively. Phylogenetic analyses were conducted using MEGA version 3.1 program [14] using the minimum evolution algorithm with the Kimura 2-parameter model.

## 3. Results

### 3.1. Case Description

**Figure 1:**
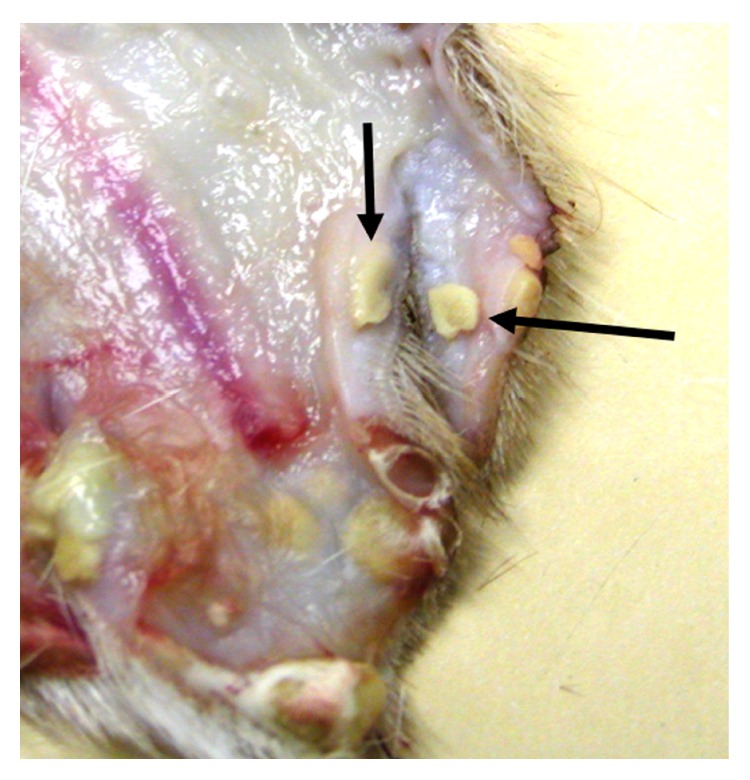
Gross presentation showing multifocal tan cutaneous nodules (arrows) containing numerous *Demodex* mites.

**Figure 2:**
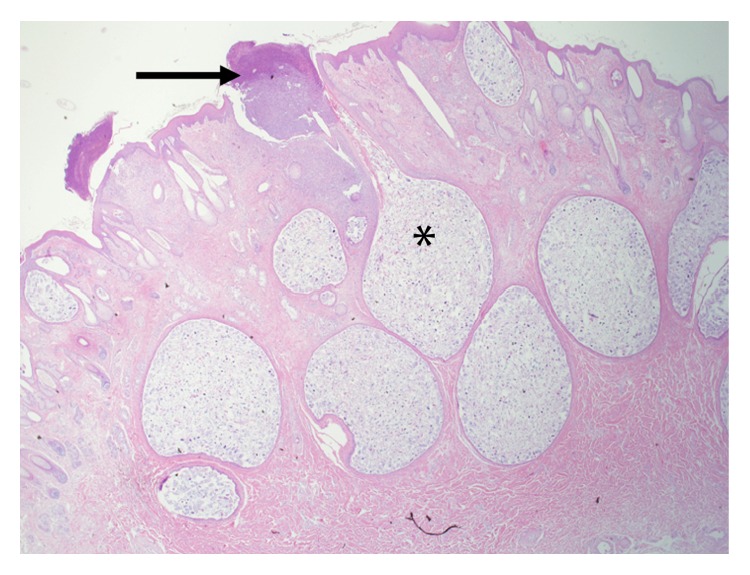
Multiple cystic structures filled with extremely high numbers of mites (∗). High accumulation of eosinophils in the area of serocellular crusting (arrow).

**Figure 3:**
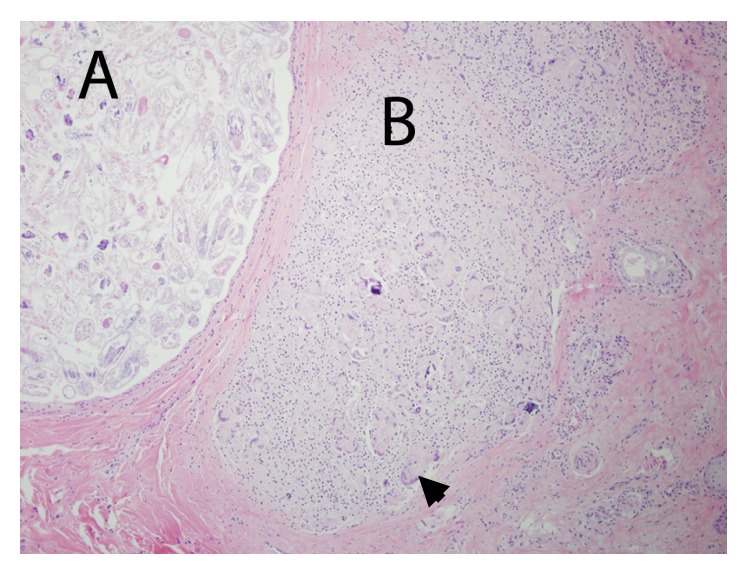
(A) Cystic structure filled with mites and no inflammation present. (B) Ruptured cyst with mite remnants and multinucleated giant cells (arrowhead).

**Figure 4:**
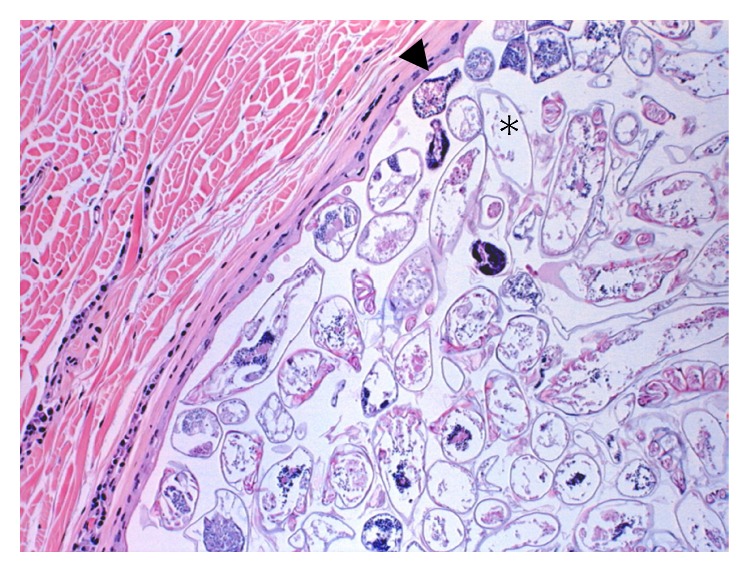
Wall of a single cyst filled with *Demodex* mites (∗) showing the stratified squamous epithelium (arrowhead) and lack of inflammatory response around the cyst.

**Figure 5:**
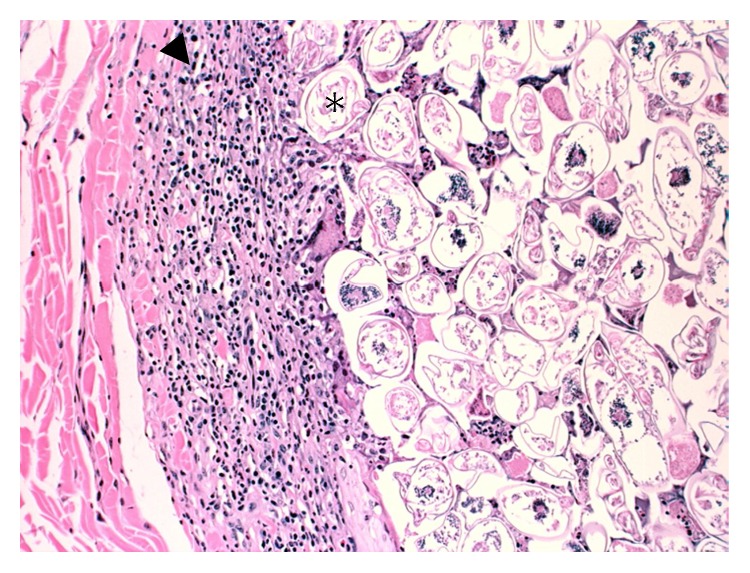
Disruption of this cyst filled with *Demodex* mites (∗) which has resulted in an inflammatory infiltration (arrowhead).

 The deer weighed 58 kg and was in good physical condition with adequate fat deposits around the kidneys and within the mesentery. The only gross lesion noted during necropsy was the presence of multiple ~1-2 cm tan cutaneous nodules on the head, legs, and lateral aspects of the thorax and abdomen, but no alopecia was noted (Figure 1). The nodules, when examined microscopically, were shown to contain several hundreds to thousands of intrafollicular *Demodex* mites (Figures 2 and 3). Histologically, hair follicles were extremely enlarged and the cysts were lined by stratified squamous epithelium varying between three and more than 20 cells in thickness (Figure 4). The majority of follicles were not surrounded by inflammatory cells (Figures 2, 3, and 4), but some cysts were disrupted resulting in infiltration with lymphocytes, plasma cells, and eosinophils (Figure 5). Skin samples also displayed a perifollicular lymphocytic to granulomatous dermatitis with scattered eosinophils. Marked fibrosis was noted around some affected hair follicles. Occasional giant granulomas with lymphocytes and scattered eosinophils were noted.

### 3.2. Morphology

**Figure 6:**
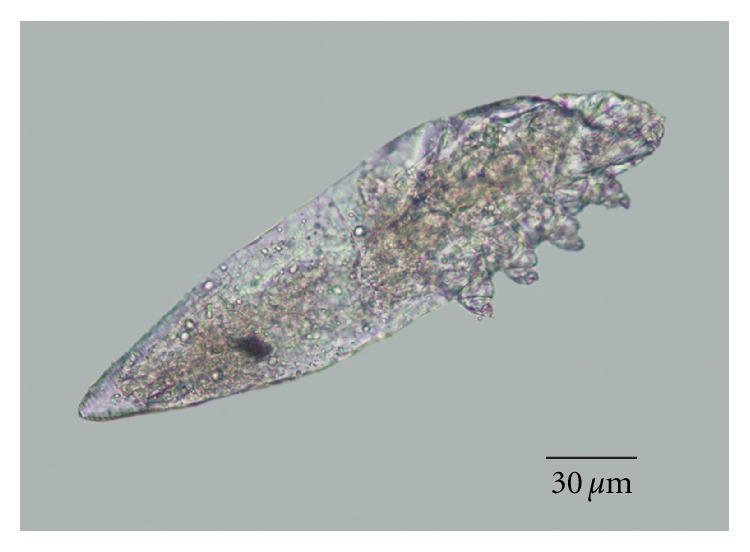
Oil mounted single mite obtained from the clinical case.

**Figure 7:**
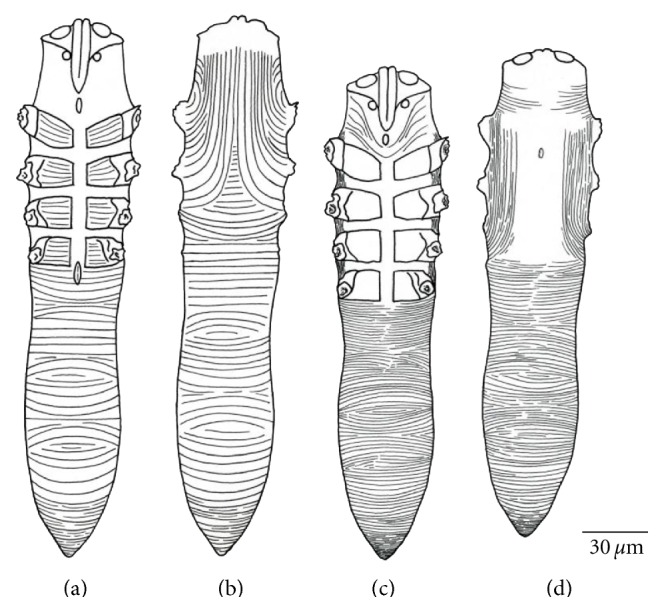
Line drawings of the *Demodex* sp. from white-tailed deer. Female ((a) ventral; (b) dorsal) and male ((c) ventral; (d) dorsal).

The *Demodex* sp. from the white-tailed deer in this study was most similar morphologically to *D. kutzeri* and *D. acutipes* from European red deer [3, 12] and is easily differentiated from *D. odocoilei* from white-tailed deer [4] (Figures 6 and 7). Numerous morphological measurements of larval, nymphal, and adult mites can be used to differentiate the *Demodex* sp. from *D. odocoilei* from white-tailed deer (Tables 1 and 2). This *Demodex* sp. can also be differentiated from *D. acutipes* and *D. kutzeri* of red deer by some measurements of adult mites (e.g., width of podosoma and opisthosoma and length of aedeagus) (Table 2). 

### 3.3. Molecular Characterization

**Figure 8:**
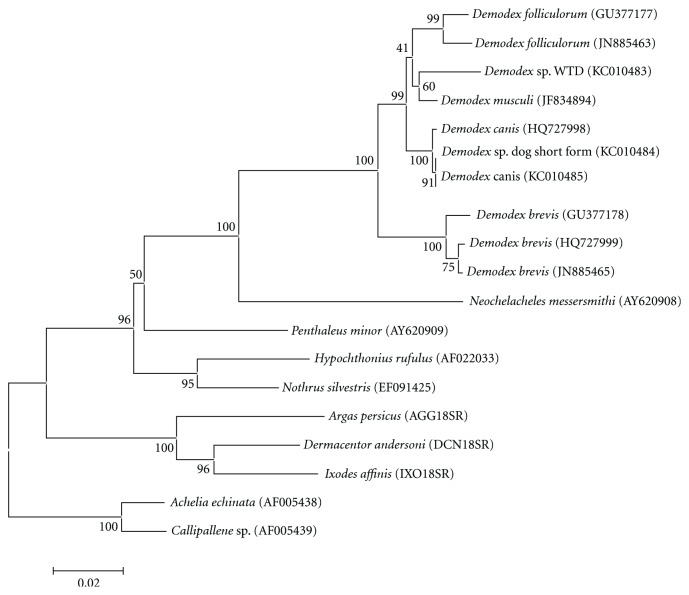
Phylogenetic relationships of *Demodex* species with related mites. The *Demodex* sp. of WTD (white-tailed deer) and the two *Demodex* spp. samples obtained from dogs in the current study are bolded. *Demodex ursi* from the black bear was excluded because the sequence was shorter than other sequences.

Overlapping sequences obtained from the *Demodex* sp. of WTD, *Demodex canis, *and the *Demodex *sp. (short form) from the dog were 1,035, 1,029 and 1,020 bp, respectively. The two canine *Demodex* sequences only differed by a single base and differed from the *Demodex* from the WTD by 34 bp. The 766 bp obtained from *D. ursi* from the black bear was identical to the overlapping sequence of the *Demodex* sp. of WTD. The final alignment of the *Demodex* spp. from the WTD and dogs with related organisms (shown in Figure 8) was 1,093 bp; 405 bp was variable and of those 267 was parsimonious informative. The samples from the genus *Demodex* formed a monophyletic clade with *D. brevis* from humans at the base. The *Demodex* sp. of WTD formed a monophyletic clade with *D. musculi* from laboratory mice (JF834894) and these were in a clade with *D. folliculorum* from humans (Figure 8). This clade was a sister clade to various *D. canis* samples. Basal to the *Demodex* clade was *Neochelacheles messersmithi*, another mite in the superfamily Chelyetoidea, which inhabits spore tubes of polypore fungi and preys on astigmatic mites [15]. The separation of *Demodex* spp. from *N. messersmithi* was well supported (bootstrap 100%). 

## 4. Discussion and Conclusions

Since 1977, 16 cases of demodicosis in white-tailed deer have been documented at SCWDS (unpublished data), and when combined with previously documented cases, *Demodex*-infested white-tailed deer have been detected throughout the host range (Maine, South Dakota, Texas, and numerous southeastern and Midwestern states) [4, 5, 7, 8, 16–18]. In only a few of these cases were the mites morphologically determined, and most were identified as *D. odocoilei* [4], but recently, three deer were infested with *D. kutzeri* [5]. Infection of individual mammalian species with multiple species of *Demodex* is common (e.g., three species in domestic dogs, two in domestic cats); thus the finding of a third *Demodex* species infesting white-tailed deer is not unexpected. The *Demodex *sp. identified in this clinical case was on average 30% larger than *Demodex odocoilei* but was morphologically similar to *D. acutipes* and *D. kutzeri*, both parasites of red deer (*Cervus elaphus*) from Europe, and the latter was recently reported from three species of cervids from North America [5]. However, the *Demodex* sp. can be differentiated from both by several morphologic measurements. 

 Similar to the initial description of *D. odocoilei* [4], the *Demodex *sp. in this paper inhabits the hair follicles and sebaceous glands of the white-tailed deer. Similar to observations with *D. odocoilei*, little to no inflammation was associated with the majority of hair follicles and sebaceous glands containing the mites. However, in contrast to *D. odocoilei*-infected deer, the *Demodex* sp.-infected deer had hair follicles that were severely distended and contained thousands of mites within giant cystic, follicular structures. The gross presentation was more similar to that described for *D. kutzeri* and *D. acutipes* from red deer in Europe [3, 12] and *D. kutzeri* from three species of cervids from Colorado, South Dakota, and South Carolina [5]. The case material used in this study was obtained from a clinical case submission of a noncaptive hunter-killed deer; therefore, we were unable to investigate the chronicity of lesions associated with the *Demodex* sp. in white-tailed deer. Similar to previous reports of *D. kutzeri* in cervids, the current clinical case presented with nodular demodicosis which tends to be rare in cases of *D. odocoilei* infestation [5].

 Based on phylogenetic analyses, the *Demodex* sp. from the white-tailed deer was most similar to *D. musculi* from laboratory mice. Interestingly, *Demodex* from deer, dogs, mice, and humans (*D. folliculorum*) formed a clade separate from the other human-infesting *Demodex* species (*D. brevis*) (current study and [11]). The monophyletic *Demodex* group was related to *N. messersmithi*, another member of the superfamily Chelyetoidea supporting the classical relationships of this superfamily of mites and other recent studies on the phylogenetics of the Chelyetoidea [15, 19]. Use of the 18S rRNA gene was useful in distinguishing most species in the current study and one previous study on human and canine species [11]. However, based on partial 18S rRNA gene sequence, we were unable to distinguish the mites from white-tailed deer and bear as different *Demodex* species; however, *Demodex ursi* from black bears [20] and the *Demodex* sp. from white-tailed deer are morphologically distinct. In the future, *Demodex* sequence data of more variable gene targets from additional species of *Demodex* obtained from a diverse set of hosts will allow studies on the diversity of *Demodex* sp. infecting different host species as well as studies on host specificity. 

## Figures and Tables

**Table 1:** Measurements of ova, larvae, and nymphs of *Demodex* species from cervids.

	*Demodex* sp.	*D*. *kutzeri* ^b^	*D*. *odocoilei* ^c^
Ovum			
Length (*μ*m ± SD)	65.1 ± 4.1 (*n* = 38)^a^	74 ± 6.2	59.1 ± 3.5
Width	38.7 ± 3.8	50 ± 5.5	26.9 ± 3.7
Larva length	124.9 ± 11.6 (*n* = 38)	121.4 ± 11.1	93.5 ± 11.2
Nymph length	205.1 ± 19.4 (*n* = 40)	253.7 ± 38.8	167.8 ± 17.9

^a^ *n*: the number of each stage measured.

^b^See [3].

^c^See [4].

**Table 2:** Morphologic measurements of adult *Demodex* species from cervids.

	Male				Female			
	*Demodex* sp. from WTD (*n* = 44)^b^	*D*. *kutzeri* ^c^	*D*. *acutipes* ^d^	*D*. *odocoilei* ^e^	*Demodex* sp. from WTD (*n* = 42)^b^	*D*. *kutzeri*	*D*. *acutipes*	*D*. *odocoilei*
Gnathosoma								
Length^a^	29.4 ± 3.4	25.5 ± 1.0	24.7 ± 1.5	15.8 ± 1.1	31.5 ± 3.9	31.5 ± 1.3	27.7 ± 1.2	18.1 ± 0.8
Width	31.8 ± 3.4	30.0 ± 1.6	35.4 ± 1.7	16.9 ± 0.9	32.9 ± 3.07	32.5 ± 2.1	40 ± 2.1	19.3 ± 0.8
Podosoma								
Length	70.4 ± 5.8	64.7 ± 5.1	75.9 ± 2.2	46.7 ± 2.9	71.9 ± 5.4	66.8 ± 3.0	87.1 ± 2.8	53 ± 2.1
Width	56.7 ± 4.3	68.3 ± 6.9	53.7 ± 3.8	30.7 ± 1.5	56.6 ± 5.2	69.4 ± 4.2	61.2 ± 4.2	35.3 ± 1.6
Opisthosoma								
Length	110.2 ± 12.3	126.0 ± 12.0	91.8 ± 9.8	86 ± 12.7	123.5 ± 12.9	144.2 ± 14.1	105.3 ± 7.4	98.7 ± 13.5
Width	49.9 ± 6.2	66.9 ± 4.3	45.1 ± 2.9	23.9 ± 1.8	45.3 ± 6.0	63.0 ± 6.5	53.4 ± 3	27.8 ± 2.2

Total length	209.1 ± 13.1	216.1 ± 13.9	192 ± 10.8	148.4 ± 13.8	225.5 ± 13.4	242.5 ± 15.2	220.2 ± 9.5	169.8 ± 13.6

Aedeagus	29.8 ± 0.9	32.6 ± 2.7	23.1 ± 0.8	17.7 ± 0.8				
Vulva					11.2 ± 2.0	10.7 ± 0.6	~9–12	4.6 ± 0.4

^a^Lengths and widths are given in *μ*m and are followed by ± SD.

^b^ *n*: the number of each stage measured.

^c^See [3].

^d^See [12].

^e^See [4].
